# On the function of redfronted lemur’s close calls

**DOI:** 10.1007/s10071-012-0507-9

**Published:** 2012-05-10

**Authors:** Femke J. Pflüger, Claudia Fichtel

**Affiliations:** 1 Behavioral Ecology and Sociobiology Unit, German Primate Center, Kellnerweg 4, 37077 Göttingen, Germany; 2 Courant Research Centre “Evolution of Social Behaviour”, University of Göttingen, Göttingen, Germany

**Keywords:** *Eulemur rufifrons*, Contact calls, Group cohesion, Social interaction, Social cognition

## Abstract

In order to maintain group cohesion, many social mammals and birds regularly produce close calls. In some primate species, close calls appear to have a dual function: calls addressed at a broad class of targets serve to maintain group cohesion, whereas the same calls directed at a specific target serve to regulate subsequent social interactions. Redfronted lemurs (*Eulemur rufifrons)* produce different types of close calls: grunts, long grunts, hoos and meows. In order to study the function of these calls, we conducted focal observations and vocal recordings from eight adult males and females out of four social groups in Kirindy Forest, Western Madagascar. Redfronted lemurs produce long grunts, hoos and meows at relatively low rates during foraging, resting or group movements, respectively. Grunts were given most often and more or less constantly during foraging and traveling. Calling rate increased when the risk of separation increased and may thus promote group cohesion. Grunts given during approaches of other group members resulted more often in friendly interactions than approaches that were not accompanied by a grunt. Thus, redfronted lemurs produce specific but also generic contact calls, whereas the latter calls have a dual function that varies depending on the addressed audience: they act as an auditory beacon to maintain group cohesion and serve as signals of benign intent to avoid costly conflicts and facilitating social interactions.

## Introduction

Many animals are organized into permanent social groups. Living in groups has many benefits, including reduced individual predation risk, joint resource defense, cooperative foraging, shared vigilance and information transfer (Alexander 1974; Bertram 1978; van Schaik 1983; Zemel and Lubin 1995). However, living in a group also results in inter-individual conflicts and costs, such as competition over resources and mates or increased pathogen transmission. These factors limit the size of groups and act as a centrifugal force on group cohesion (Alexander 1974; Bertram 1978). In order to maintain group cohesion and social stability despite these conflicts, individuals need to regulate spacing between group members and employ mechanisms to reduce conflicts (Aureli and de Waal 2000; Radford and Ridley 2008).

Many animals produce vocal signals that appear to be involved in the maintenance of group cohesion and decision-making processes before collective movements (Boinski and Garber 2000; Fichtel and Manser 2010). Several species produce the so-called close calls when traveling, foraging and resting (Struhsaker 1967; Boinski and Garber 2000; Rendall et al. 1999; Radford 2004; Trillmich et al. 2004; Koda et al. 2008). These calls may serve as a ‘location marker’ to announce the caller’s spatial position but also to regulate spacing between group members. For example, group-living pied babblers (*Turdoides bicolor*) produce chucks during foraging to maintain cohesion, but also to regulate spacing of potential competitors (Radford and Ridley 2008).

In primates, many species produce long distance calls to regulate intra- and inter-group spacing over longer distances but also close distance calls (Snowdon et al. 1983; Biben 1993; Cheney and Seyfarth 1996; Fichtel and Kappeler 2002; Digweed et al. 2007). Close distance calls appear to be addressed at several recipients and in many species such as golden lion tamarins (*Leonthopithicus rosalia*), white-faced capuchin monkeys (*Cebus capucinus*) and Japanese macaques (*Macaca fuscata*) the rate of calling increased when the risk of becoming separated from the group was high as during foraging or moving or in dense habitats (Boinski 1993; Boinski et al. 1994; Koda et al. 2008; Ey et al. 2009). Interestingly, the function of the same close calls seems to vary when they are addressed at a specific target. For example, baboons grunt when they move and forage but also when approaching mothers attempting to inspect or handle their young infants (Rendall et al. 1999). The likelihood of a subsequent peaceful interaction was usually higher when approaches were accompanied by grunts. Thus, during social interactions, baboon grunts seem to facilitate subsequent peaceful behavior among interaction partners (Cheney et al. 1995).

A similar function of close calls has been suggested in other species such as stumptailed macaques (*M. arctoides;* Bauers 1993) and Japanese macaques (Masataka 1989). In rhesus macaques (*M. mulatta*), the likelihood of an aggressive interaction was directly associated with the use of grunts and girneys. Females were much less likely to initiate aggression when approaches at lower ranking females were accompanied with contact calls than when they remained silent (Silk et al. 2000). In this context, these calls have been suggested to function as generic commitments signaling what animals will do next (Silk 2002; but see Whitham et al. 2007). Thus, close calls appear to have depending on the audience at which they are addressed a dual function: they may either function to maintain group cohesion or to regulate social interactions (Fichtel and Manser 2010).

While most previous studies of close calls were performed on anthropoid primates, data from a greater variety of taxa could provide important comparative information for a more comprehensive understanding of the function of close calls in group-living primates. Lemuriformes, which are relatively small-brained (Barton 1996), form an independent primate radiation (Tattersall 1982) and represent the most primitive group-living primates (Bearder 1987; Richard 1987). Furthermore, group living in Malagasy primates evolved at least twice independently (Kappeler 1999). During millions of years of isolation, they converged with other group-living primates only in the most fundamental ways, but deviate in several aspects of their social organization, such as group size or sex ratio (van Schaik and Kappeler 1993; Kappeler 1997; Erhart and Overdorff 2008). Thus, a broad comparative perspective including the best living models of the earliest gregarious primates can enrich reconstructions of primate social behavior and cognition (Fichtel and Kappeler 2010).

Redfronted lemurs (*Eulemur rufifrons*) are group-living lemurs with a complex vocal repertoire. They produce several calls during group movements, foraging but also during social interactions as well as alarm calls (Pereira and Kappeler 1997; Fichtel and Kappeler 2002). In particular, grunts are more or less constantly produced while moving, foraging and during social interactions (Pereira and Kappeler 1997) and may, thus, serve to maintain group cohesion and to regulate social interactions. However, they also produce other calls such as hoos, meows and long grunts while resting and moving, indicating that redfronted lemurs may use several types of close calls.

In order to examine the function of these calls, that is grunts, long grunts, meows and hoos, we investigated their usage in four social groups of wild redfronted lemurs in Kirindy forest, Western Madagascar. If these calls serve to maintain cohesion, we predicted that they should be produced more often when the risk of separation increases and the group is widespread, exhibiting low cohesion as during foraging and moving. If these calls also serve to regulate social interactions, we predicted that they are also produced during social interactions and that the likelihood of aggression decreases when approaches are accompanied by a close call.

## Materials and methods

### Study area and subjects

In this study, we observed 16 adult individuals out of four groups of redfronted lemurs (*E. rufifrons*) in Kirindy forest, Western Madagascar (Kappeler and Fichtel 2012a). Because groups of redfronted lemurs consist on average of 2–3 females (Kappeler and Fichtel 2012b), we chose two adult females and two adult males in each group as focal animals to have a balanced sample size. As part of an ongoing long-term study, all animals are individually marked with nylon collars or radio transmitters.

### Data collection

Focal animals (eight females, eight males) were observed using continuous sampling (Altmann 1974) during 30-min observation sessions. Data collection included continuous recordings of the focal animal’s vocalizations and a documentation of the general activity such as group movements between food patches and resting sites (GM), locomotion during foraging or approaching conspecifics (LO), feeding (FE), resting (RE) and resting in social interactions (SI) (see Table 1 for definitions). Recordings of vocalizations were made with a Marantz PMD 670 CF-Recorder and a Sennheiser ME 80 directional microphone.

**Table 1:** Definitions of behavioral patterns modified from Pereira and Kappeler (1997)

Group movements	Movements of the whole group on the ground or in trees for at least 4 min or at least 15 m between food patches and resting sites (Pyritz et al. 2010)
Locomotion	Short distance movements while foraging, approaching conspecifics or departure from them. Short distance movements were defined as movements lasting between 10 s and 4 min
Feeding	Searching with nose over ground or terminal branches, manually grasping, biting or chewing potential food items, including feeding movement for less than 10 s
Resting	Individual remains inactive for at least 1 min
Social resting	Resting in body contact or huddling with one or more group members for at least 1 min
Approach	An animal moved from beyond a distance of at least 3 m to a distance of less than 50 cm to a targeted individual, including subsequent affiliative or agonistic interactions between the animals
Affiliative behavior	*Grooming* was defined as repeated strokes over partner’s pelage using the toothcomb and/or tongue. Two animals *huddle* together when at least two animals rest in a hunched position, keeping less than one-third of body-to-body contact on the resting partner. *Sitting near* was defined as resting within 0.3 m of a group member
Agonistic behavior	*Submission* was defined as self-displacement when the target immediately moved for more than 1 m away from the approaching animal *Aggressions* included biting, cuffing, grabbing and chasing

Additionally, all social interactions between the focal animal and another group member were noted. During approaches, we recorded whether focal animals produced contact calls when they entered within a 3 m radius of the targeted animal. We also documented whether the target showed affiliative or agonistic behavior toward the approaching individual.

### Analysis of call rates

**Fig. 1:**
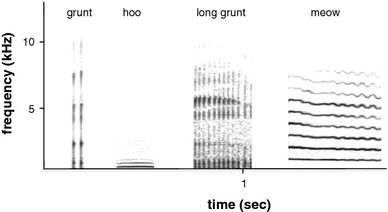
Spectrograms of a grunt, hoo, long grunt and meow. All spectrograms were generated in Avisoft-SASLabPro Software (Avisoft Bioacoustics; frequency resolution: window length = 200 ms, bandwidth = 70 Hz, resolution: 47 Hz, temporal resolution: overlap = 87.5 %, 1/bandwidth = 14.2 ms, resolution = 2.67 ms)

Vocalizations were recorded for 100 h and were digitized at a sampling rate of 48 kHz (16-bit resolution) in Avisoft—SASLab Pro software (Avisoft Bioacoustics). Several recordings were discarded due to high levels of background noises produced by cicadas, reducing the recording duration to 57 h. This resulted in an unbalanced data set per individual. In order to have an equal observation time of about 4 h for each focal animal, only 12 (six adult males and six adult females) animals were included in the analysis of call rates. Only calls of high-quality recordings were included in the analysis, resulting in 16.425 calls. We categorized vocalizations as grunts (*N* = 14,825), long grunts (*N* = 303), hoos (*N* = 396) and meows (*N* = 120; Fig. 1). Calls given in response to predators, during aggressive interactions, or between group communication, that is chucks, woofs, chutter and croaks (Fichtel and Kappeler 2002), were summarized as other calls (*N* = 907) and were only included in the comparison of call rates across call types. For each individual, we calculated the call rate of each call type across contexts. To compare call rates across call types and the use of certain call types across activities, we calculated generalized linear mixed effects models (GLMM) and linear mixed effects models (LMM) with square-root transformed response variables and REML estimation (Zuur et al. 2009; Bolker et al. 2009). The response variable was the rate of the different call types. Activity (i.e., group movement, feeding, locomotion, resting, social resting) and sex were included as fixed factor, and individuals nested within social groups were included as random factors to account for non-independence of repeated measurements of individuals (e.g., Zuur et al. 2009). We used maximum likelihood ratio tests to test the final model with fixed factors against the null model including only the intercept and random factors (Faraway 2006). For the LMM, we used Markov chain Monte Carlo methods to generate* p* values (Bates et al. 2008). Tukey post hoc comparisons were conducted with the multcomp package (function glht in R; Hothorn et al. 2008).

### Analysis of social interactions

For the analysis of social interactions, we included interactions between all adult focal individuals (*N* = 16) and their adult or subadult group mates (defined as a dyad). In the four social groups, 82 of dyads were possible, but only 55 of them occurred during observation. In order to test the hypothesis that close calls serve to mediate social interactions, we used a multiple logistic regression with binomial error (link = logit; e.g., Bolker et al. 2009). The affiliative or agonistic outcome of an approach was the dichotomous response variable. As fixed factor we used whether the approach was accompanied with a grunt or not. To exclude the possibility that results were influenced by other factors besides the production of grunts, sex (three levels: dyads of two males (mm), two females (ff) or a male and a female (mf), age of the approaching animal (adult or subadult) and kin were also included as fixed factors. Since repeated observations of the same dyad do not provide independent information, dyads nested within groups were defined as a random factor in the model (e.g., Zuur et al. 2009). Statistical tests were computed in R 2.13.0 (R Developmental Core Team 2011).

## Results

### Usage of call types across contexts

**Fig. 2:**
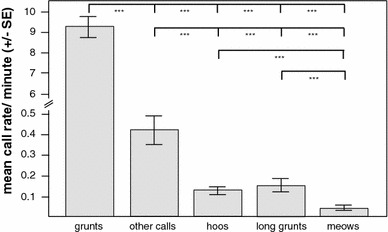
Mean rates (±SE) of grunts, hoos, long grunts and meows. Tukey post hoc comparison: ***p < 0.001, **p < 0.01, *p < 0.05

Grunts were the most frequently emitted call type and were more often produced than all other call types (Fig. 2; Table 2, GLMM, χ^2^ = 15,599, *df* = 4, *p* < 0.001). In comparison with grunts hoos, long grunts, meows and other calls were produced at relatively low rates. Call rates differed between other calls and hoos, long grunts and meows and between hoos and meows as well as between long grunts and meows (Fig. 2).

**Table 2:** Estimates, SE and* p* values of the models for (a) call rates (b) grunt rates, (c) hoo rates, (d) meow rates (e) long grunt rates and (f) social interactions (f = female, m = males)

Response variable	Random factor	Fixed factors	Estimate	SE	*p* value
(a) Call rates	ID nested in group	Intercept	−0.98	0.13	<0.001
		Grunt	3.13	0.06	<0.001
		Hoo	−1.83	0.13	<0.001
		Long grunt	−1.11	0.13	<0.001
		Meow	−2.38	0.22	<0.001
(b) Grunt rate	ID nested in group	Intercept	2.23	0.23	<0.001
		Group movements	1.49	0.21	<0.001
		Locomotion	2.29	0.17	<0.001
		Resting	−0.64	0.15	<0.001
		Social resting	−0.50	0.21	<0.05
		Sex	−0.33	0.29	0.26
(c) Hoo rate	ID nested in group	Intercept	0.04	0.03	0.23
		Group movements	0.13	0.05	<0.01
		Locomotion	0.07	0.04	0.07
		Resting	0.19	0.04	<0.001
		Social resting	0.08	0.05	0.12
		Sex	−0.03	0.03	0.32
(d) Meow rate	ID nested in group	Intercept	0.02	0.03	0.39
		Group movements	0.18	0.03	<0.001
		Locomotion	0.01	0.02	0.61
		Resting	0.02	0.02	0.32
		Social resting	0.03	0.03	0.41
		Sex	−0.01	0.02	0.61
(e) Long grunt rate	ID nested in group	Intercept	0.13	0.04	<0.01
		Group movements	0.14	0.05	<0.01
		Locomotion	0.14	0.04	<0.01
		Resting	0.07	0.04	0.07
		Social resting	0.02	0.05	0.70
		Sex	−0.12	0.05	<0.01
(f) Social interactions	Dyad nested in group	Intercept	−4.93	1.44	<0.001
		Grunt (no)	3.79	0.73	<0.001
		Sex (mf)	0.13	1.3	0.92
		Sex (fm)	0.56	1.34	0.68
		Sex (ff)	1.46	1.38	0.29
		Kin	1.27	0.7	0.07
		Age	1.84	0.7	<0.01

**Fig. 3:**
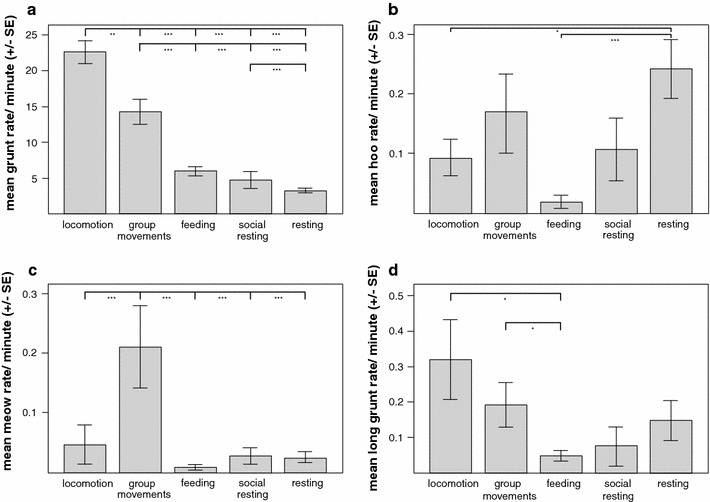
Mean rates (±SE) of **a** grunts, **b** hoos, **c** meows and **d** long grunts across behavioral contexts. Tukey post hoc comparison: ***p < 0.001, **p < 0.01, *p < 0.05

Grunts were significantly more often produced during locomotion and group movements, but less often emitted during feeding, resting and social resting (Fig. 3a; Table 2, LMM, χ^2^ = 301.76, *df* = 5, *p* < 0.001). The grunt rate decreased from locomotion over group movements, feeding and social resting to resting. Sex did not influence grunt rates.

Hoos were given significantly more often during resting than locomotion and feeding, but hoo rates differed not between group movements, feeding and social resting (Fig. 3b; Table 2, LMM, χ^2^ = 2106.4, *df* = 5, *p* < 0.001). Sex had no effect on hoo rates.

Meows were significantly more often produced during group movements than in other contexts (Fig. 3c; Table 2, LMM, χ^2^ = 2818.4, *df* = 5, *p* < 0.001). The rate of meows was not influenced by sex.

Long grunts were given significantly more often during locomotion and group movements than during feeding, but long grunt rates differed not between feeding, social resting and resting (Fig. 3d; Table 2, LMM, χ^2^ = 2057.8, *df* = 5, *p* < 0.001). Sex had a significant effect on the rate of long grunts with males producing long grunts more often than females (Table 2).

### Social interactions

**Fig. 4:**
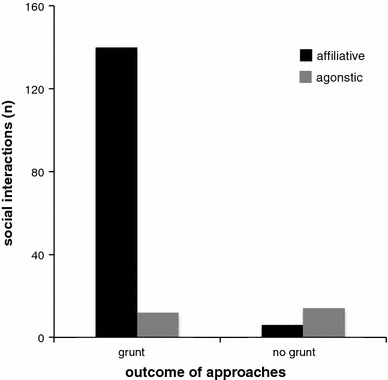
Frequency of affiliative and agonistic behavior depending on whether approaches were accompanied by grunts or no grunts

We observed 172 social interactions. In 152 of these, the approaching animal grunted. The probability of an affiliative interaction was higher (92 %) when the approaching animal produced grunts than without producing grunts (30 %) (Table 2; Fig. 4). Age also had an effect on the outcome of an approach with approaching adults receiving aggression less often (9 % of approaches) than subadults (28 % of approaches) (Table 2), but the interaction between grunting and age was not significant. Other fixed factors like sex and kin did not influence the behavior of the receiver (Table 2).

## Discussion

In this study, we investigated the function of redfronted lemur’s close calls: grunts, long grunts, hoos and meows. The grunt was the most often produced call type. Redfronted lemurs produced them more or less constantly in all contexts, but most often in contexts when the risk of separation from the group was high. Grunts were also produced during social interactions and seem to facilitate peaceful interactions. Thus, grunts appear to have a dual function and may serve as a location marker to maintain group cohesion but also as a signal of benign intent. Hoos, meows and long grunts were produced at relatively low rates in comparison with grunts but, interestingly, in rather specific contexts: hoos are given most often during resting, meows during group movements and long grunts during locomotion and group movements. Thus, redfronted lemurs use a combination of generic and context-specific close calls.

### Dual function of grunts: maintenance of cohesion and signals of benign intent

According to the definition of Rendall et al. (2000), redfronted lemurs’ grunts can be classified as contact calls because they are relatively quite calls given at high rates while the group moves or forages and the risk of becoming separated is therefore high. Grunts were the most frequently produced call type and their rate increased from resting over feeding to locomotion. These results are in line with the usage of contact calls in birds and other anthropoid primates, which also produce close calls at a higher rate when the risk of becoming separated from group members increases (Radford and Ridley 2008; Suguira 2007; Koda et al. 2008; Ey et al. 2009). Thus, from the sender’s perspective, grunts appear to signal the senders’ location. However, if grunts indeed may modulate a receiver’s tendency to approach or to avoid individuals, is less well understood. Hence, the proximate mechanism driving the close function is not entirely clear, and further playback experiments are required to elucidate the receiver’s perspective in this context (Fichtel and Manser 2010).

In social interactions, redfronted lemurs usually grunt while approaching conspecifics and the probability of subsequent aggressive behavior decreased when approaches were accompanied by a grunt. As in baboons and macaques (Bauers 1993; Cheney et al. 1995; Silk et al. 2000), redfronted lemurs may use grunts to communicate their intention to behave peacefully toward others. Contrary to the societies of baboons and macaques, redfronted lemurs exhibit a rather egalitarian social structure with only one male being dominant over other males, no dominance relationship among females and a lack of female dominance (Pereira et al. 1990; Ostner and Kappeler 1999). Consequently, redfronted lemurs are limited in reducing costly conflicts through defined dominance ranks. Since approaches accompanied with grunts resulted more often in affiliative interactions involving grooming, and grooming represents a mechanism to reduce conflicts (Port et al. 2009), the emission of a benign vocal signal might be an even more so important commitment tactic to avoid conflicts. Since high stress levels may have long-term consequences on an individuals’ fitness (e.g., Beehner et al. 2005), mechanisms to avoid conflicts are pivotal. Female baboons (*Papio ursinus*) that received grunts at high frequencies from dominant females had indeed lower glucocorticoid levels (e.g., Crockford et al. 2008). Hence, the use of a benign signal appears to be a crucial mechanism to avoid conflicts and subsequently to reduce stress.

Interestingly, redfronted lemurs responded aggressively in only 16 % of approaches, and, as in other primates aggression was more often directed toward subadults (Pereira and Fairbanks 2002). Although these low agonistic rates reflect the general low agonistic rate in redfronted lemurs (Ostner and Kappeler 1999; Erhart and Overdorff 2008; Pyritz et al. 2011), they may also reflect the effectiveness of using benign vocal signals to avoid conflicts. Another mechanism to reduce conflicts is reconciliation, which has been reported in many anthropoid primates (Aureli and de Waal 2000). Because redfronted lemurs also reconcile soon after a conflict to reduce the probability of further attacks (Kappeler 1993), similar mechanisms of avoiding conflicts have evolved in strepsirrhine and anthropoid primates.

### Function of long grunts hoos and meows

Long grunts, which are grunts of longer duration than normal grunts (Pereira and Kappeler 1997), are given at relatively low rates but most often while moving. Hoos and meows were in comparison with grunts produced at relatively low rates and in rather specific contexts: hoos during resting and meows during group movements. Hoos are very soft tonal calls, and usually one individual started to produce a hoo whereas one by one others replied with a hoo while continuing to rest. Because in this context redfronted lemurs might be less vigilant, the production of poorly localizable low calls might be advantageous to avoid being discovered by predators through their own vocalizations (Ryan et al. 1981; Fichtel 2009). Meows are also tonal calls but in contrast to hoos higher in frequencies and much louder. Because meows were mainly produced during group movements, when the group is widespread, it might be advantageous to produce a specific call that travels over longer distances to maintain cohesion. Also, closely related ringtailed lemurs (*Lemur catta)* produce a similar call to maintain cohesion over longer distances (Oda 1996). Thus, as in pygmy marmosets (*Cebuella pygmea*), the different forms of close calls may degrade differently in the habitat and might be used differentially as a function of how close they were to other conspecifics (de la Torre and Snowdon 2002). However, further studies on the acoustic characteristics of the habitat and degradation of these vocalizations are required. Because other group-living mammals and birds, such as African elephants (*Loxodonta africana*), bottlenose dolphins (*Tursiops truncates*), several parrots (*Amazona auropalliata*, *A. albifrons*, *Aratinga canicularis*, *Brotogeris jugularis*) black-billed gulls (*Larus bulleri*), or green woodhoopoes (*Phoeniculus purpureus*), also produce close calls in the context of maintaining group cohesion (Evans 1982; Poole et al. 1988; Janik and Slater 1998; Bradbury 2003; Radford 2004), acoustic signals are a widespread mean in communicative networks to facilitate maintenance of group cohesion and coordination processes (Fichtel and Manser 2010).

In summary, we showed that redfronted lemurs use a combination of context-specific and generic close calls. Grunts, as generic close calls, have a dual function that depends on the audience at which the call is directed. Grunts that appear to be addressed at several targets seem to serve in the maintenance of cohesion, whereas grunts that are addressed at specific targets may serve to signal the benign intent of the approaching animal. Signals of benign intent are low-cost signals of strategic commitment and provide recipients with reliable evidence about the actor’s intention and disposition and are effective in facilitating social interactions (Silk et al. 2000; Silk 2002).

This finding is of particular interest, because group-living in Malagasy primates evolved independently (Kappeler 1999) and they converged with other primates only in the most fundamental ways. However, with regard to mechanisms of conflict management (signals of benign intent and reconciliation; Kappeler 1993), they exhibit comparable complexity as anthropoid primates. Interestingly, lemur’s abilities in the domain of social cognition appear to deviate from the better-known anthropoid primates (Fichtel and Kappeler 2010), but recent studies on social learning and the evolution of behavioral traditions (Hosey et al. 1997; Kendal et al. 2010; Fichtel and Kappeler 2011; Schnoell and Fichtel 2012) as well as the results of this study indicate that their degree of social organization and their cognitive abilities in the social domain are not as limited as previously thought (Jolly 1966; Deaner et al. 2006; Fichtel and Kappeler 2010).
